# Rapid and low-cost screening for single and combined effects of drought and heat stress on the morpho-physiological traits of African eggplant (*Solanum aethiopicum*) germplasm

**DOI:** 10.1371/journal.pone.0295512

**Published:** 2024-01-30

**Authors:** Vincent A. Opoku, Michael O. Adu, Paul A. Asare, Justice Asante, Godswill Hygienus, Mathias N. Andersen

**Affiliations:** 1 Department of Crop Science, School of Agriculture, College of Agriculture and Natural Sciences, University of Cape Coast, Cape Coast, Ghana; 2 Department of Agroecology, Faculty of Technical Sciences, Aarhus University, Tjele, Denmark; Hainan University, CHINA

## Abstract

Drought and heat are two stresses that often occur together and may pose significant risks to crops in future climates. However, the combined effects of these two stressors have received less attention than single-stressor investigations. This study used a rapid and straightforward phenotyping method to quantify the variation in 128 African eggplant genotype responses to drought, heat, and the combined effects of heat and drought at the seedling stage. The study found that the morphophysiological traits varied significantly among the 128 eggplants, highlighting variation in response to abiotic stresses. Broad-sense heritability was high (> 0.60) for chlorophyll content, plant biomass and performance index, electrolyte leakage, and total leaf area. Positive and significant relationships existed between biomass and photosynthetic parameters, but a negative association existed between electrolyte leakage and morpho-physiological traits. The plants underwent more significant stress when drought and heat stress were imposed concurrently than under single stresses, with the impact of drought on the plants being more detrimental than heat. There were antagonistic effects on the morphophysiology of the eggplants when heat and drought stress were applied together. Resilient genotypes such as RV100503, RV100501, JAMBA, LOC3, RV100164, RV100169, LOC 3, RV100483, GH5155, RV100430, GH1087, GH1087*, RV100388, RV100387, RV100391 maintained high relative water content, low electrolyte leakage, high Fv/Fm ratio and performance index, and increased biomass production under abiotic stress conditions. The antagonistic interactions between heat and drought observed here may be retained or enhanced during several stress combinations typical of plants’ environments and must be factored into efforts to develop climate change-resilient crops. This paper demonstrates improvised climate chambers for high throughput, reliable, rapid, and cost-effective screening for heat and drought and combined stress tolerance in plants.

## 1.0 Introduction

African eggplant (*Solanum aethiopicum*) is an essential traditional vegetable consumed and cultivated in most African countries [1]. The crop is a vital genetic resource for building resilience against biotic [2] and abiotic stress [3, 4]. African eggplant production provides livelihood to approximately 60% of resource-poor farmers [5], with many beneficiaries along its value chain, including vendors, most of whom are women. Compared to other vegetables, African eggplant is vital for nutrition (rich in potassium, magnesium, calcium, zinc, iron and essential vitamins), food security purposes and income generation for smallholder farmers [6]. The role of African eggplant in achieving Sustainable Development Goal 2 (SDG 2) of ending hunger, achieving food security and improved nutrition, and promoting sustainable development of agriculture is significant. Nonetheless, African eggplant is primarily cultivated in sub-Saharan Africa, a region highly vulnerable to climate change [7]. Hence, the growth and yield of the African eggplant, like other crops, is affected by heat and drought, leading to a yield gap of over 78% between actual and potential yields [6, 7].

Despite much insight into the single effect of drought and heat [9], crops are often subjected to the simultaneous occurrence of these stressors under field conditions [9–11]. Thus, drought and heat stress are interlinked, with the combined stress causing significant impacts on yield and economic outcomes than individual stressors. Combined drought and heat stress adversely alter plant phenology, morphology, and physiology, including growth, chlorophyll content, leaf photosynthesis, plant water content and grain yield [12, 13]. These stressors can also harm reproductive development, reducing flowering, fertilisation, and sink capacity [14, 15].

Plants respond to drought and heat through various adaptive mechanisms [9, 16–19]. Researchers have screened for heat and drought tolerance in various crops, such as cotton, cabbage, maize, tomatoes, and amaranth [17, 20–25], by examining underlying traits like water content, photosynthesis, chlorophyll, biomass, specific leaf area, root growth, stomatal conductance, and water use efficiency. Decreased electrolyte leakage, increased water content, chlorophyll content, biomass, specific leaf area, improved photosynthesis, membrane stability, and other mechanisms explain tolerance [11, 26, 27]. However, the mechanisms vary between plant species [28]. Phenotyping for tolerance is challenging due to the complexity of stressors and a limited understanding of adaptive mechanisms among plant species [29].

Several studies have focused on developing elite genotypes for crops such as chilli pepper [30], amaranth [23], tomatoes [25], and eggplant [31]. However, there is a lack of research investment in African eggplant, making it a neglected vegetable (Aguessy et al., 2021) despite its potential as a means of subsistence. For a more holistic assessment, it is critical to study the response of African eggplants to combined heat and drought stress (Raja et al., 2020). In this study, we aimed to investigate the mechanism of combined drought and heat stress tolerance in African eggplant germplasm by examining the physiological and morphological responses of plants at the seedling stage using locally built improvised growth chambers and provide recommendations for genetic materials to be included in the breeding of drought and heat-tolerant genotypes.

## 2.0 Materials and methods

### 2.1 Environmental conditions

**Fig 1:**
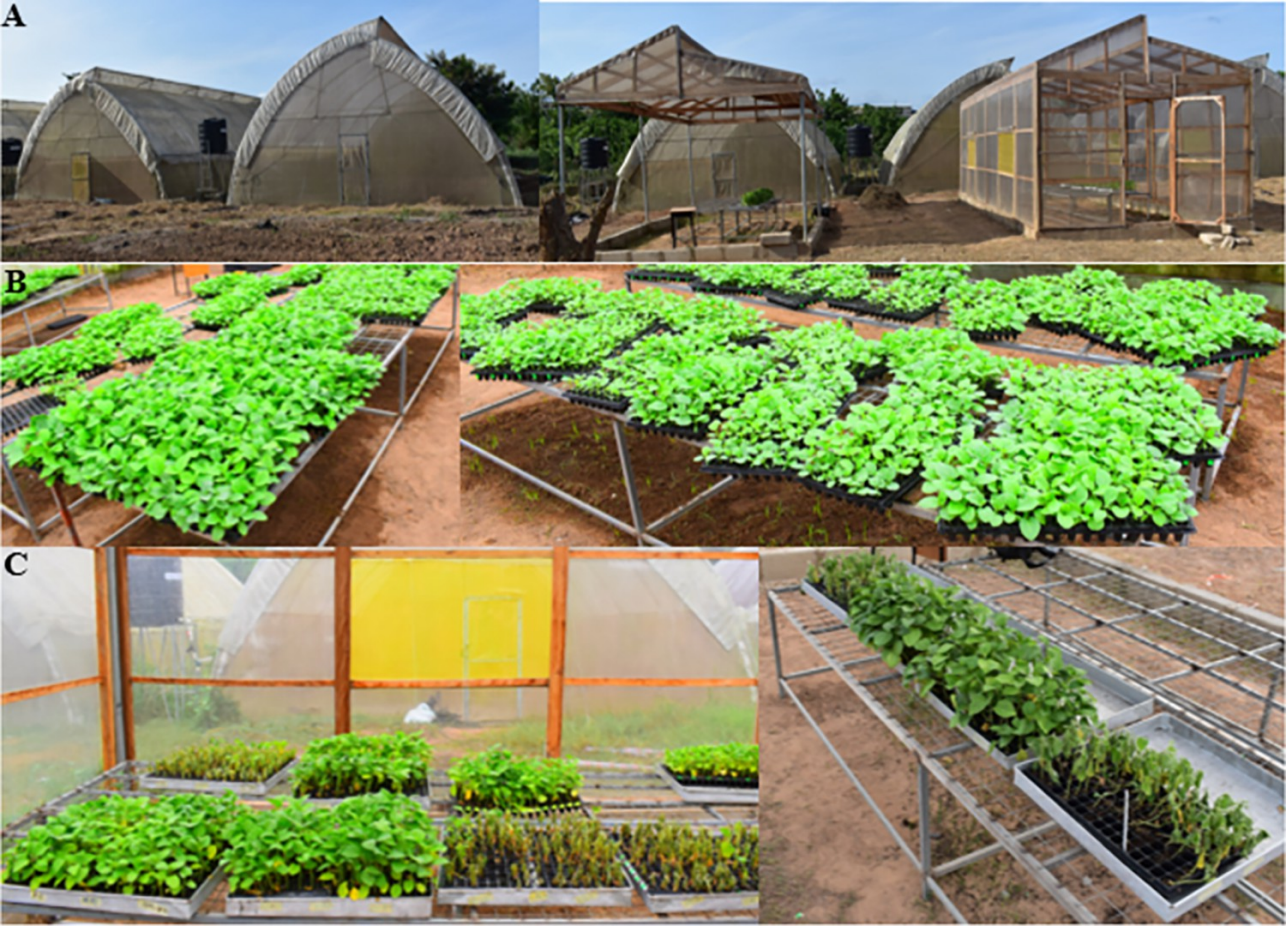
Image of **(A)** Locally constructed growth chambers. Ambient growth chamber with open sides and heat chamber with a yellow ventilation mesh net. **(B)** Eggplant genotypes showed consistent growth after germination at the nursery stage in the greenhouse. **(C)** Stressed plants in ambient and heat chamber after four (4) days of stress exposure. Genotypes exposed to concurrent heat and drought on the right side and genotypes under drought stress on the left side, each with their respective control treatments.

The study was conducted at the A.G Carson Technology Centre of the School of Agriculture, University of Cape Coast (UCC) (5.1155°N, 1.2909°W) in the Central Region of Ghana. A sawtooth plastic film conventional greenhouse and two locally-built growth chambers were used as improvised climate chambers. The temperature in the traditional greenhouse ranged between 19 and 30°C, and the humidity was between 70–90%. The growth chambers were constructed with stainless steel poles and a 150 μm thick transparent plastic film polythene covering that allowed ∼ 86% solar transmission (Fig 1A). The ambient chamber was roofed with a transparent plastic polythene film; however, the facade and other sides of the structure were open. The second improvised climate chamber was enclosed with transparent plastic film polythene to accumulate heat. It had a door built with a wooden frame and transparent plastic polythene film. A vent was covered with a plastic yellow mesh on the roof, and 1 × 1m ventilation windows with foldable plastic flap window blinds on both sides of the design to help regulate the temperature within the chamber (Fig 1A). The enclosed growth chamber provided a temperature-controlled environment of ∼10°C above the open growth chamber. Genotypes were grown for one month in a traditional greenhouse with temperatures ranging between 19–30°C and humidity between 70 and 90%. One month after germination, plants were subjected to a mean temperature of 28/19°C and 41/32°C Day/Night and mean relative humidity of 70 and 50% for plants grown in the open and enclosed chamber, respectively (S1 Fig). When the temperature in the heat chamber reached 60°C, most often during mid-day, the ventilation window was opened to allow fresh air into the heat chamber (Fig 1A) to maintain the maximum temperature at approximately 50°C. Both chambers’ microclimatic (temperature and relative humidity) were monitored and logged at 20-minute intervals using a data logger (WS-520 indoor/outdoor thermometer and humidity, Denver Electronics A/S). Control plants were watered by sitting nursery trays in metallic pans for 1 hour in both control and heat chambers to avoid drought stress.

### 2.2 Genetic materials

A total of 128 African eggplant genotypes, with 114 genotypes obtained from the World Vegetable Center Genebank (https://genebank.worldveg.org/), were used for the study. The selected African eggplant genotypes were maintained at the World Vegetable Center Genebank but mainly from West African countries, including Benin, Burkina Faso, Cote d’Ivoire, Ghana, Nigeria, and Togo (S1 Table). The remaining 14 genotypes were obtained from the Ghana Council for Scientific and Industrial Research (CSIR-Fumesua) and local (LOC) agrochemical and seed shops within the study area. This panel of African eggplant had not previously been evaluated for heat and drought stress tolerance.

### 2.3 Experimental design and treatment

Two independent trials, laid in an alpha lattice design, were used. Owing to the high number of genotypes, rapid screening of the eggplant accessions was performed in eight batches. Each batch comprised 16 genotypes, laid in two independent blocks and 16 plants per block. A common genotype (LOC 4) was included in each screening batch. Variation between trials was assessed from the common genotype and accounted for as a cofactor but was observed as insignificant. The first trial was conducted from April to July, and the second was from August to October 2021. Factors for the study were genotypes and stress, which included no stress, drought, heat, and combined heat-drought stress.

### 2.4 Sowing, watering, and management

Five-week seedlings (6–8 leaves) were initially raised from seeds in the conventional greenhouse before being transferred to the locally-constructed climate growth chambers. Nursery trays (128 cells; 28 x 54 cm with cells; 5 × 3 cm) were uniformly filled with Fertiplus universal potting soil (Dizengoff Ghana, https://www.baltoncp.com/) and topped with Poly-Feed™ (https://www.haifa-group.com) applied at 40 g per 16 L knapsack sprayer. Poly-Feed^TM^ was applied to seedlings twice before imposing stress treatment and ceased two weeks before and for data collection. For the first five weeks, in the conventional greenhouse, two healthy seeds of each genotype were hand-sown approximately 2 cm below the surface of the potting mix. The seedlings were thinned to one stand per cell seven days after germination. To irrigate the potting mix, the nursery trays were placed in water-filled basins and allowed to saturate by capillarity for approximately one hour, after which the basins were emptied, and pots were allowed to drain off excess water [32] (Fig 1C). Nursery trays were rotated in the conventional greenhouse to avoid possible effects of temperature, light, and humidity fluctuations or gradients.

### 2.5 Rapid screening and imposition of stress

Seedlings were transferred from a conventional greenhouse to locally constructed climate-growth chambers. Plants were divided into two groups for each genotype: half were kept in an ambient (open) growth chamber, and the other half were kept in a heat (enclosed) growth chamber. In each chamber, plants of each genotype were further divided into two groups. In the heat chamber (closed), the first group of plants for each genotype was watered throughout the experimental period to experience only heat stress. In contrast, in the other group, watering was progressively ceased to expose plants to concurrent heat-drought stress.

Similarly, in the ambient chamber (open), each genotype’s first group of plants was watered throughout the experimental period to serve as a non-stress (control) group. In contrast, the other group was exposed to progressive drought effects, which served as drought stress. For each treatment, there were 16 plants per genotype in a nursery tray, and each 128-cell tray contained eight genotypes. There were two replicate trays for each genotype in each chamber. Drought and heat stress lasted for five days in the chambers. The stress and non-stress trays were rotated regularly in the ambient and heat chambers to eliminate possible gradients and variations in temperature and radiation.

### 2.6 Data collection

Data on the morphophysiological parameters of stress and non-stress genotypes were collected on the 1^st^, 2^nd^, and 4^th^ days after stress imposition. A total of 5 plants per treatment combination were sampled destructively for data collection on each sampling day. The length and breadth of two randomly selected fully opened leaves were measured with a meter rule. The leaf area was calculated as described by Carvalho et al. [33]. The product of the length and breadth of the leaf was multiplied by a factor of 0.75. Shoot fresh weights were measured using an electronic scale. The leaves of each genotype were carefully separated from the shoot and measured to obtain fresh leaflet weight. Samples were oven-dried at 80°C for three days when they attained constant weight to determine the shoots’ and leaflets’ dry weight. The total leaf area and dry leaf weight were used to calculate the specific leaf areas. Leaf mass per area was estimated as a quotient of 1 and a specific leaf area [18].

Leaf chlorophyll content was measured nondestructively using a meter (SPAD-502 Plus Chlorophyll Meter, Spectrum Technologies, Inc., Aurora, IL, USA). The chlorophyll content was measured from three randomly selected leaves per plant, and the average was estimated to represent the plant. Leaf chlorophyll fluorescence (ratio of variable fluorescence by maximum fluorescence or Fv/Fm ratio) and performance index were measured with a PEA portable fluorometer (Hansatech Instruments Ltd.), with a detection time of 1 s and emitting light at a wavelength of 650 nm, with an intensity of 3500 μmol m^-2^s^-1^ [34]. Before measuring chlorophyll fluorescence, two (2) leaves per plant were dark-adapted for 30 minutes using a leaf clip. The Fv/Fm ratio and performance index measurement were taken on the third most fully expanded leaflet, avoiding the midrib section [35].

Relative leaf water content was measured by clipping two young leaves from each plant selected for sampling, from which four-leaf discs were made using a number 9-cork borer (1.9 cm diameter). The weight of the fresh disc was recorded, after which the discs were placed in a petri dish containing enough water to float the leaf discs. The petri dish was allowed to stand for 24 hours at room temperature to ensure full hydration of the leaf disc. The hydrated leaf discs were gently removed, blotted with a paper towel, and weighed to obtain the turgid weight of the disc. The leaf discs were then oven-dried at 80°C for 24 hrs. The dry weight of the leaf disc was recorded. The equation described by Jensen et al. (2020) [36] was used to calculate relative leaf water content.


$$Relative\;leaf\;water\;content=\frac{Fresh\;disc\;wt-Dry\;disc\;weight}{Turgid\;weight-dry\;disc\;weight}\times 100$$ [Eq 1]

Electrolyte leakage from leaves was measured using the procedures of Hatsugai and Katagiri (2018) [37] with slight modification in the volume of deionised water used. Three leaves per seedling were washed with deionised water and perforated into a disc with a radius of 5.5 mm. Each disc was placed in a tube containing 10 mL of deionised water and left overnight at room temperature. Subsequently, the initial conductivity of distilled water was measured using a Eutech PC 450 electrical conductivity meter. The tube was heated in a boiling water bath of 100°C for 30 minutes, after which samples were allowed to cool at room temperature and final conductivity was recorded. Electrolyte leakage was calculated as the initial and final conductivity quotient and expressed as a percentage.

### 2.7 Statistical analysis

Statistical analysis was performed using analysis of variance (ANOVA) in R with built-in packages such as lme4 and lmerTest [38, 39]. Data collected on morpho-physiological parameters required no transformation since the data were normally distributed after exploration. Means for various experimental factors, including genotype, sampling day, stress type, and trial, were separated using Tukey’s test of significance (LSD; p < 0.005). Principal Component Analysis (PCA) was performed using the measured traits under contrasting stress conditions to elucidate the contribution of various traits to tolerance under various stress conditions. The Kaiser criterion was used to identify essential components, with any element having an eigenvalue of >1 retained [40, 41]. Similarly, correlation analysis was performed among the measured traits under control, drought, heat, and combined heat and drought conditions. PCA and correlation were performed with the functions of prcomp from the built-in R stats package [42]. The results were visualised using the factoextra package [43], FactoMineR and ggbiplot2 [44]. Ward’s hierarchical approach was used for cluster analysis based on the minimum variance linking method with Euclidean distance [45]. The optimal number of clusters was chosen based on the ‘elbow criterion’, which compares the Sum of Squared Differences (SSD) for different cluster solutions [46]. A biplot and factor map were used to summarise the variation between genotypes and the contribution of each cluster membership to traits that confer tolerance under contrasting stress (heat, drought, and combined heat and drought) conditions. The residual maximum likelihood (REML) procedure was employed to determine variance components to estimate broad-sense heritability (H^2^) across the experiments for the remaining parameters. In the REML analyses, all factors were categorised as random, allowing us to assess the proportional contribution of genotype to overall trait variation [47]. The random model used (Eq 2) used for REML analyses were

$$Y_{ijk}=\mu +g_{i}+s_{j+}t_{k}+d_{l}+gb_{ij}+{Ꜫ}_{ijkl}$$ [Eq 2]

where *Y*_*ijk*_ represents the observation from *ijkl*^*th*^ genotype, stress type, trial, and sampling day; *μ* is the overall mean; *g*_*i*_ *is* the effect of the *i*^*th*^ genotype; *s*_*j*_ is the effect of the *j*^*th*^ stress type; *t*_*k*_ is the effect of the *k*^*th*^ trial; *d*_*l*_ is the effect of the *l*^*th*^ sampling day; *gs*_*ij*_ is the interactive effect of the *i*^*th*^ genotype with *j*^*th*^ stress type, and *Ꜫ*_*ijkl*_ is the experimental error.

We estimated broad-sense heritability (H^2^) as the quotient of the genotypic variance and the total phenotypic variance for the trait ($\sigma_{g}^{2}/\sigma_{p}^{2}$) [48]. We employed Eq 3, as applied by [49], to estimate the phenotypic variance, where r is the number of replicates, n is the number of trials, and $\sigma_{g}^{2}$ × t is the genotype × trial variance.


$$\sigma_{p}^{2}=\frac{\sigma_{g}^{2}\times t}{n}+\frac{\sigma_{Ꜫ}^{2}}{rn}$$ [Eq 3]

## 3.0 Results

### 3.1 Growth of seedlings, summary statistics, analysis of variance, and broad-sense heritability

Eggplant genotypes performed well for up to 46 days in the nursery and locally constructed climate growth chambers. During the experimental period, there were no observable signs or symptoms of pest attack and nutrient deficiencies (Fig 1B). Plant growth was uniform, and there was no etiolation in the nursery greenhouse or chambers. The canopy of plants was well developed, with most genotypes forming more than five leaves five weeks after germination (Fig 1B).

The coefficient of variation (CVs) was low (5.1–19.7%) for Fv/Fm ratio, shoot fresh weight, shoot dry weight electrolyte leakage, and relative water and chlorophyll content. The CVs were moderate (23–39.5%) for performance index, total leaf area, specific leaf area, and leaf mass per area (Table 1). Broad-sense heritability (H^2^) estimates of measured traits were low to high and ranged from 0.3 to 0.8 (Table 1). Broad-sense heritability (H^2^) was high (> 0.60) for chlorophyll content, plant biomass and performance index, electrolyte leakage, and total leaf area. The relative leaf water content, Fv/Fm ratio, and leaf mass per area had an intermediate (0.3–0.6) H^2^, but specific leaf area recorded low H^2^ (< 0.30) (Table 1). Both genotypic and phenotypic variance components were low (0–10%) for Fv/Fm ratio, intermediate (10–20%) for electrolyte leakage and chlorophyll content but high (≥ 20%) for shoot dry weight, leaf mass per area and performance index (Table 1).

**Table 1:** Descriptive statistics and broad-sense heritability for 128 eggplant genotypes screened for heat and drought tolerance at the seedling stage.

Trait (Abbreviation)	Unit	Descriptive statistics			Variance component		Broad-sense heritability
		Mean	SD	CV%	Phenotypic	Genotypic	
Electrolyte leakage (EL)	%	29	5	16.5	13.3	21	0.64
Fv/Fm ratio (Fv/Fm)	-	0.69	0.1	4.9	3.1	6	0.5
Chlorophyll content (CC)	-	31.7	4	9.3	20	33	0.6
Rel. leaf water content (RLC)	%	70.5	5	7	29	36	0.8
Shoot fresh weight (SFW)	g	1.7	0.3	19.5	5	17	0.3
Shoot dry weight (SDW)	g	0.33	0.1	20	17	29	0.6
Total leaf area (TLA)	cm^2^	19.8	4.5	23	17	26	0.7
Specific leaf area (SLA)	cm^2^ g^-1^	180.5	71.2	40	10	14	0.7
Leaf mass per area	g cm^-2^	0.01	0.003	39	28	64	0.4
Performance index (PI)	-	3.15	1.02	23.1	30	109	0.3

### 3.2 Genotypic variation in traits among eggplant genotypes

The 128 eggplant genotypes varied significantly (p < 0.001) in almost all morphophysiological measurements. Electrolyte leakage ranged from 20.8 to 46.9%, with a mean of 29.1%. The highest values were recorded by RV100218, RV100168, RV100408, RV100325, RV100213, RV100237, and RV100166, whereas genotypes JAMBA, GH5155, GH3925, GH4157, RV100505, GH1087, RV100511, RV100504, and RV100460 were among the last 20 genotypes within the distribution with the least membrane leakage (S2 Table). The eggplant genotypes significantly varied (p < 0.001) in relative water content. The mean relative water content of genotypes GH5155, GH1087, GH3883, GH1087, GH5761, GH4157, JAMBA, RV100387, and RV100478 was 26.2% higher than those of RV100222, RV100252, RV100213, RV100236, RV100224, RV100394, and RV100303 which had the lowest relative water content (S2 Table). Similarly, among 128 genotypes, biomass varied significantly (p < 0.001) with high and low values of shoot dry weight of 0.41 g and 0.15 g for GH5155 and RV100394 (S2 Table). More than 2.7-fold variation was observed in both shoot fresh weight (1–2.6 g) and shoot dry weight (0.2 − 0.4 g) (S2 Table). Genotypes GH5155, GH1087*, GH3883, JAMBA, RV100504, RV100505, RV100478, RV100390, RV100511, RV100392 and LOC 1 were among the topmost 20 genotypes with higher shoot biomass (S2 Table). Chlorophyll content ranged from 23 to 40 for RV100390 and RV100503, respectively. The mean Fv/Fm ratio among eggplant genotypes was 0.69, with genotypes LOC 1, RV100511, JAMBA, RV100281, RV100183, GH5170, RV100504, GH3883, GH3925, and GH3925 within the top 10 distribution with higher Fv/Fm ratios, whereas RV100390, RV100304, RV100271, RV100213, RV100224, and RV100222 had low Fv/Fm ratios (S2 Table). A two-fold increase in total leaf area was observed when comparing the mean of the first 20 genotypes to that of the last 20 (S2 Table). The morphophysiological traits measured among eggplant genotypes at the seedling stage varied significantly (p < 0.001) between trials, except for chlorophyll content, specific leaf area, relative water content, and performance index (Table 2).

**Table 2:** Mean squares from analysis of variance for 128 eggplant genotypes screened for heat and drought tolerance at the seedling stage. Mean square with a probability significantly different at p < 0.05 (*), p < 0.01 (**), p < 0.001 (***) and non-significant (NS). Abbreviations are defined in Table 1.

Trait	Genotype	Stress	Trial	Sampling day	Genotype × Stress	Genotype × day	Genotype × trial	Stress × day	Gen. × Stress × day
EL	3517.2***	90086***	3209.6***	159610***	918***	1033.7***	131.8***	39497.6***	390.2***
Fv/Fm	0.4***	79.9***	0.2***	85.5***	0.1***	0.2***	0.002***	18.1***	0.05***
CC	2484.1***	3099.8***	3.8^NS^	9731***	273.3***	197.5***	24.6***	5115***	52.8***
RWC	4187.8***	673448***	228.9 ^NS^	425991***	957.6***	1098***	94.4***	287324.6***	427***
SFW	22.3***	1330.5***	7***	281.8***	2.8***	3***	0.4***	246***	0.7***
SDW	0.6***	26.4***	0.2***	6.5***	0.1***	0.1***	0.01***	5.3***	0.02***
TLA	3962.3***	101099***	3790.3***	64991***	591.5***	377.1***	112.2***	27335.5***	108.9***
SLA	973989.5***	2531906***	75372 ^NS^	1495078***	279589***	181744.1***	67402.5***	1085625.4***	110169***
LMA	0.002***	0.002*	0.004***	0.002***	0.0006***	0.0004***	0.0004*	0.002***	0.0003***
PI	203.5***	2570.3***	1 ^NS^	4927***	15.5***	30.6***	7.2***	735.3***	4.6***

### 3.3 Morphophysiological response of genotypes to single and combined heat and drought stress

The interaction between stress and genotype was significant (p < 0.001) for the performance index (Table 2). Genotypes LOC 2, LOC 1, JAMBA, RV100431, RV100281, GH5761, and GH5155 were within the distribution of genotypes with high-performance indices under control, heat, and drought stress. However, RV102697, RV100228, and RV100172 recorded a high-performance index under combined heat and drought conditions compared to the other stresses (S3 Table). Electrolyte leakage was higher for genotypes exposed to concurrent heat-drought stress than for those exposed to independent stresses (S3 Table); however, genotypes RV100406, RV100216, RV100211, RV100323, RV100235, RV100323, and RV100168 had the highest electrolyte leakage when exposed to drought stress alone (S3 Table). Heat stress alone led to higher electrolyte leakage among genotypes RV100206, RV100268, and RV100251, while electrolyte leakage was high for genotypes RV100160, RV100309, and RV100167 under the control treatment (S3 Table).

The interaction of stress and genotype was significant (p < 0.001) for both shoot biomass (Table 2). Genotypes GH5170, GH3883, LOC 4, GH1087*, RV100388, JAMBA, RV100431, RV100386, and LOC 1 had the highest shoot dry weight under the control treatment. RV100409, RV100504, and GH5761 were among the genotypes with high shoot dry weight under drought stress compared to the other stressors (S3 Table). Despite the direct association observed between stress and biomass production, genotypes RV100222, RV100219, RV100296, and RV10033 had a higher shoot dry mass under heat stress than the control, drought, and combined heat/drought effects (S3 Table). Shoot fresh weight was significantly higher for genotypes Jamba, LOC 3, RV100502, RV100419, RV100409, RV100296, RV100301, RV100211, and RV100210 under combined head/drought than under single drought stress (S3 Table). The genotypes RV100268, RV100290, RV100303, RV100326, Jamba, and LOC 3 had greater shoot fresh weight under heat stress than the control, drought, and heat/drought combined (S3 Table). The interaction of stress and genotype was significant was significant (p < 0.001) on chlorophyll content and electrolyte leakage (Table 2). For example, the single effect of heat-induced higher electrolyte leakage among genotypes RV100206, RV100268 and RV100251, while electrolyte leakage was high for genotypes RV100160, RV100309, and RV100167 under control treatment (S3 Table).

### 3.4 Effect of stress on measured traits

**Fig 2:**
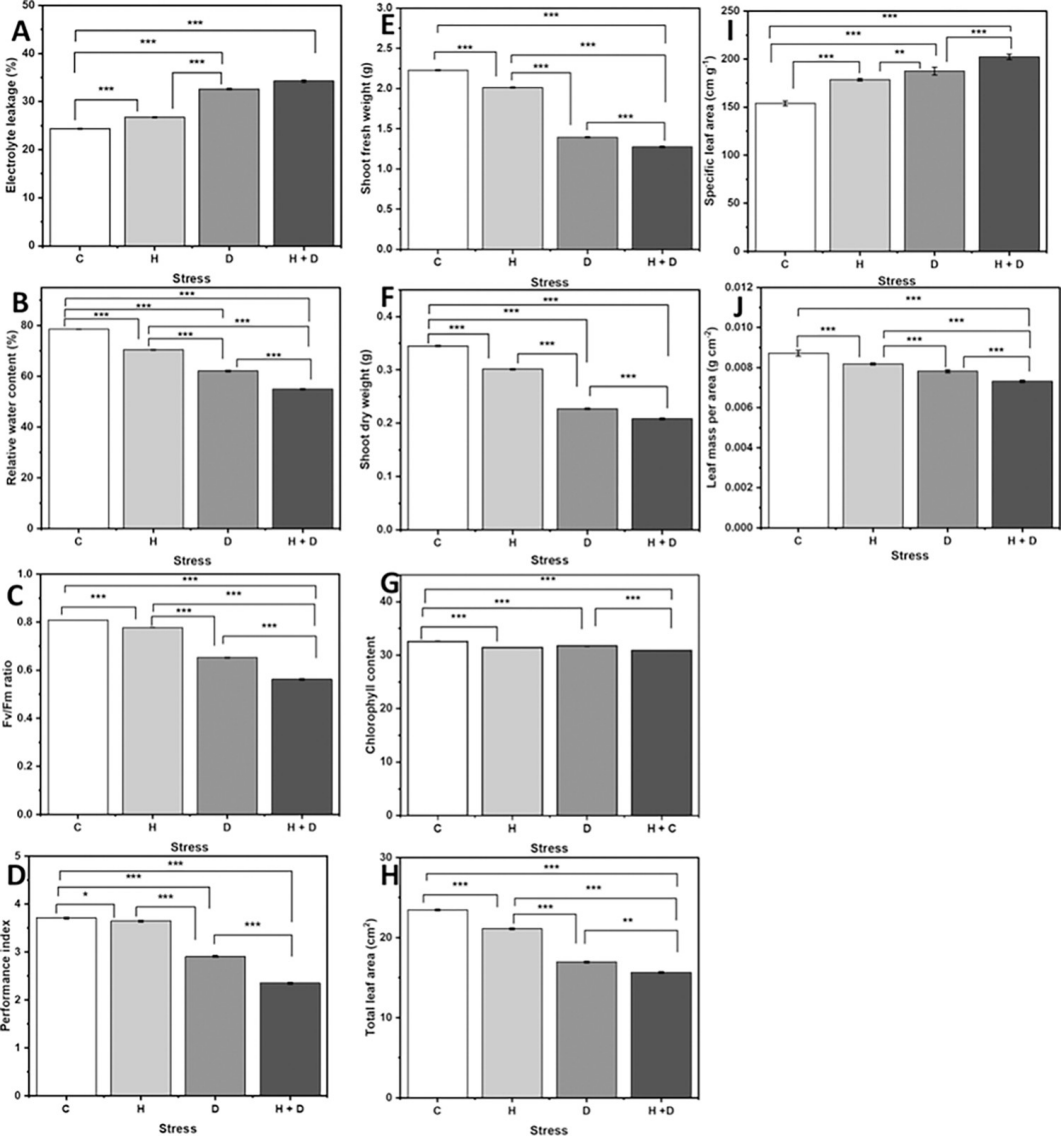
Response of 5-week-old garden egg genotypes to drought, heat or combined heat/drought. **(A)** Electrolyte leakage; **(B)** Relative leaf water content; **(C)** Fv/Fm ratio; **(D)** Performance index; **(E)** Shoot fresh weight; **(F)** Shoot dry weigh; **(G)** Chlorophyll content; **(H)** Total leaf area; **(I)** Specific leaf area and **(J)** leaf mass per area. Error bars representing the SEM Differences between stress factors and interaction between stress and day were established using ANOVA and are shown by l.s.d. (P, 0.05) bars. Where C-control, H-Heat stress, D-Drought stress and H+D-Heat and drought stress.

**Fig 3:**
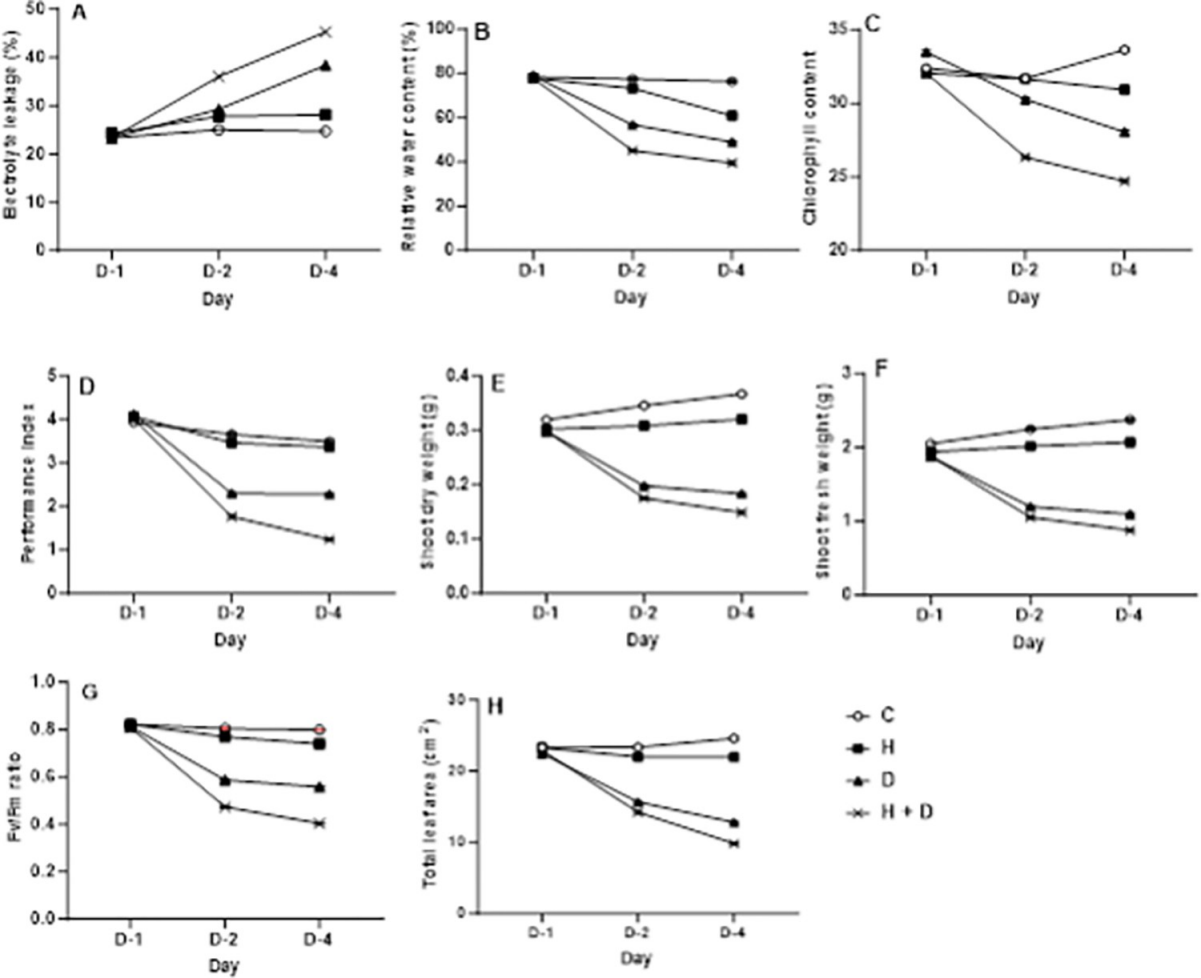
Response of 5—weeks old garden egg genotypes to stress from day 1 to 4. **(A)** Electrolyte leakage; **(B)** Relative water content; **(C)** Chlorophyll content **(D)** Performance index; **(E)** Shoot dry weight; **(F)** Shoot fresh weight; **(G)** Fv/Fm ratio; **(H)** Total leaf area. Error bars representing the SEM Differences between stress factors and interaction between stress and day were established using ANOVA and are shown by l.s.d. (P, 0.05) bars. Where C- Control, H-Heat stress, D-Drought stress and H+D-Heat and drought stress.

Relative leaf water content was reduced by 1.8, 19, and 20% when plants exposed to heat, drought, and combined stress were respectively compared to plants in control environments (Fig 2B). The effects of drought and heat stress alone resulted in a 33.9 and 9.8% increase in electrolyte leakage, respectively. However, the combined stress effect resulted in a 40.6% increase in electrolyte leakage (Fig 2A). Generally, non-stressed genotypes recorded a higher Fv/Fm ratio than stressed genotypes. However, combined heat/drought caused a significant reduction in Fv/Fm ratios compared to the single effect of heat and drought (Fig 2C). The interaction of stress and sampling days was significant for the Fv/Fm ratio and performance index (*p* < 0.001) (Table 2 and Fig 3G and 3D). Compared to the control, reduced Fv/Fm ratio levels were observed under individual and combined stress conditions (Fig 2C). A 17.9% decrease in the Fv/Fm ratio was observed under drought stress conditions. Compared to heat stress, a 16.1% decrease in Fv/Fm ratio was observed under drought stress (Fig 2C). However, the Fv/Fm ratio declined under combined heat and drought conditions (Fig 2C) with a prolonged period of stress (Fig 3G).

The individual effect of drought and heat decreased the performance index by 22% and 1.7%, respectively, compared with the control treatment. However, the combined heat and drought resulted in a 37% decrease in the performance index compared to the non-stressed plants (Fig 2D). Over time, a significant (*p* < 0.001) reduction in the performance index was observed, with a noticeable decline in drought-stressed plants on day two, a smaller decrease in heat-stressed on day four, compared to control and a reduction in plants exposed to both stresses (Fig 3D). The total leaf area of the eggplant genotypes was significantly (*p* < 0.001) affected by stress conditions (Table 2). The control, heat stress, drought stress, and combined heat and drought treatments recorded total leaf areas of 23.4 cm^2^, 23.0 cm^2^, 17.0 cm^2,^ and 15.7 cm^2^, respectively (Fig 2H). Similarly, chlorophyll content declined as the stress period was prolonged, with a significant reduction recorded on day four (Fig 3C). The combined stress effect resulted in a 5% reduction in chlorophyll content compared to drought and heat stress (Fig 2G), respectively.

### 3.5 Multivariate analysis and relationships between variables and genotypes

#### 3.5.1 Correlation between measured traits

**Fig 4:**
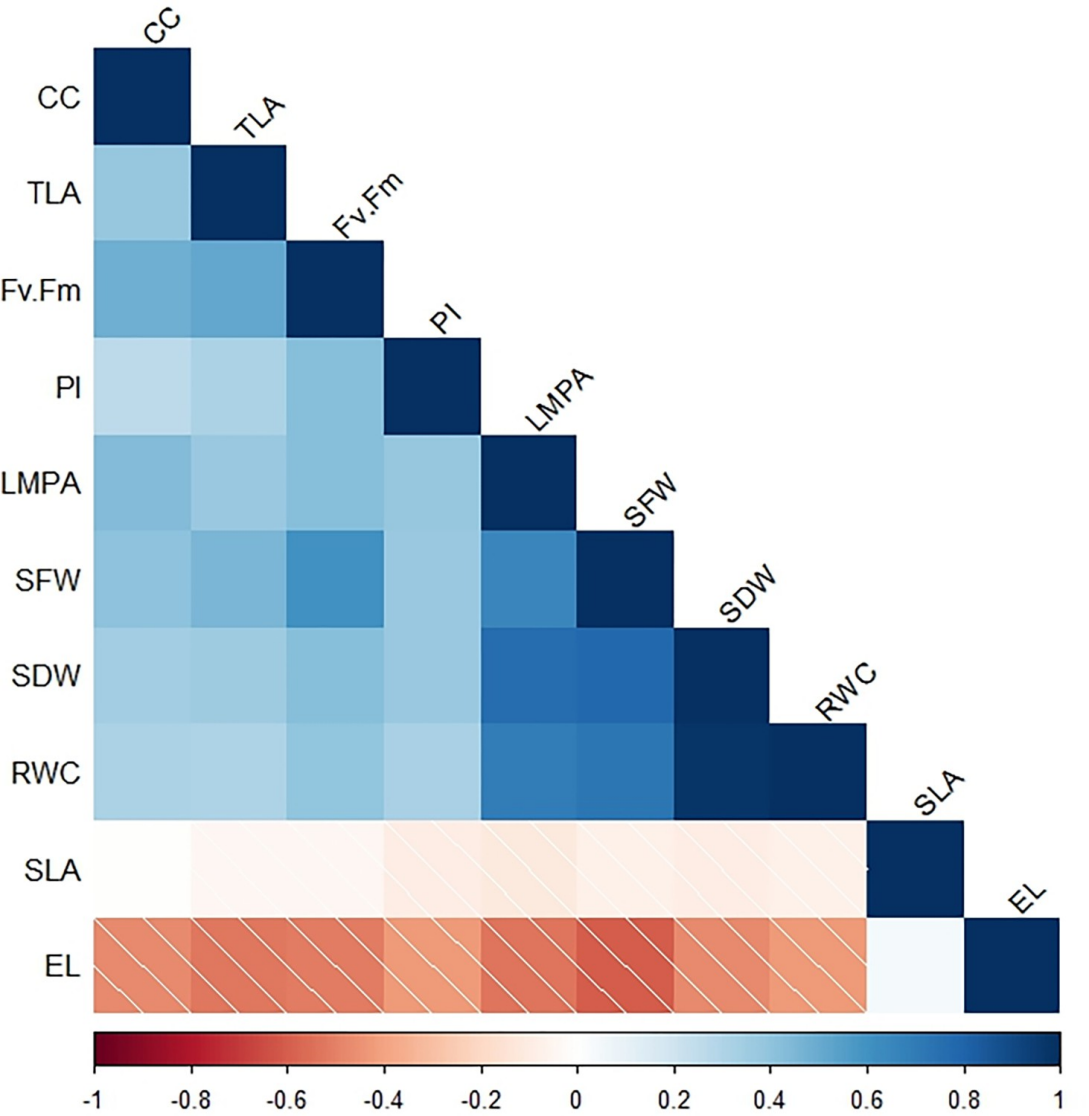
Correlations between traits observed in eggplant screened under contrasting stress conditions. The traits shown in the matrix are EL: Electrolyte leakage; CC: Chlorophyll content; PI: Performance index; SFW, shoot fresh weight; SDW: Shoot dry weight; RWC: Relative leaf water content; TLA: Total leaf area; SLA: Specific leaf area; LMPA: Leaf mass per area.

The correlation among measured traits under contrasting stress conditions exhibited similar trends. There was a strong positive relationship among many variables under stress conditions (Fig 4). Specific leaf area had a negative but insignificant (r = 0.34–0.52) correlation with biomass and physiological traits except electrolyte, which was positive (Fig 4). Similarly, electrolyte leakage had a positive and strong correlation (r = -0.78 to -0.92, p < 0.001) with the measured morphophysiological traits (Fig 4). Relative water content was strongly and positively correlated (r = 0.68 to 1, p < 0.05 or < 0.001) with biomass traits, Fv/Fm ratio, performance index, and leaf mass per area (Fig 4). Under heat stress, shoot dry weight was positively correlated (r = 0.23–0.71, p < 0.001) with chlorophyll content, Fv/Fm ratio, performance index, leaf mass per area, and shoot fresh weight (S2 Fig). Electrolyte leakage was negatively correlated (r = -0.31 and -0.34, p < 0.001) with shoot dry and fresh weight (r = -0.21 and -0.23, p < 0.05) for the performance index and chlorophyll content (S2 Fig). Electrolyte leakage was negatively correlated (r = 0.22 to -0.40, p < 0.05, 0.01 and 0.001) with shoot dry weight, chlorophyll content, shoot fresh weight, performance index, and Fv/Fm ratio under drought stress conditions (S2 Fig). Relative water content had a strong positive association with shoot fresh weight (r = 0.40, p < 0.001), shoot dry weight (r = 0.39, p < 0.001), and the Fv/Fm ratio (r = 0.21, p < 0.05) (S2 Fig). Diverse associations among the measured traits were observed under combined heat and drought conditions (S2 Fig). Relative water content had a significant negative (r = -0.35, p < 0.001) correlation with electrolyte leakage but no observable relationship with biomass parameters and photosynthetic efficiency parameters such as Fv/Fm ratio (S2 Fig). Shoot dry weight had a weak but positive significant relationship (r = 0.0.20 to 0.33, p < 0.05, and 0.01) with chlorophyll content and the Fv/Fm ratio, respectively (S2 Fig).

#### 3.5.2 Principal component analyses

**Fig 5:**
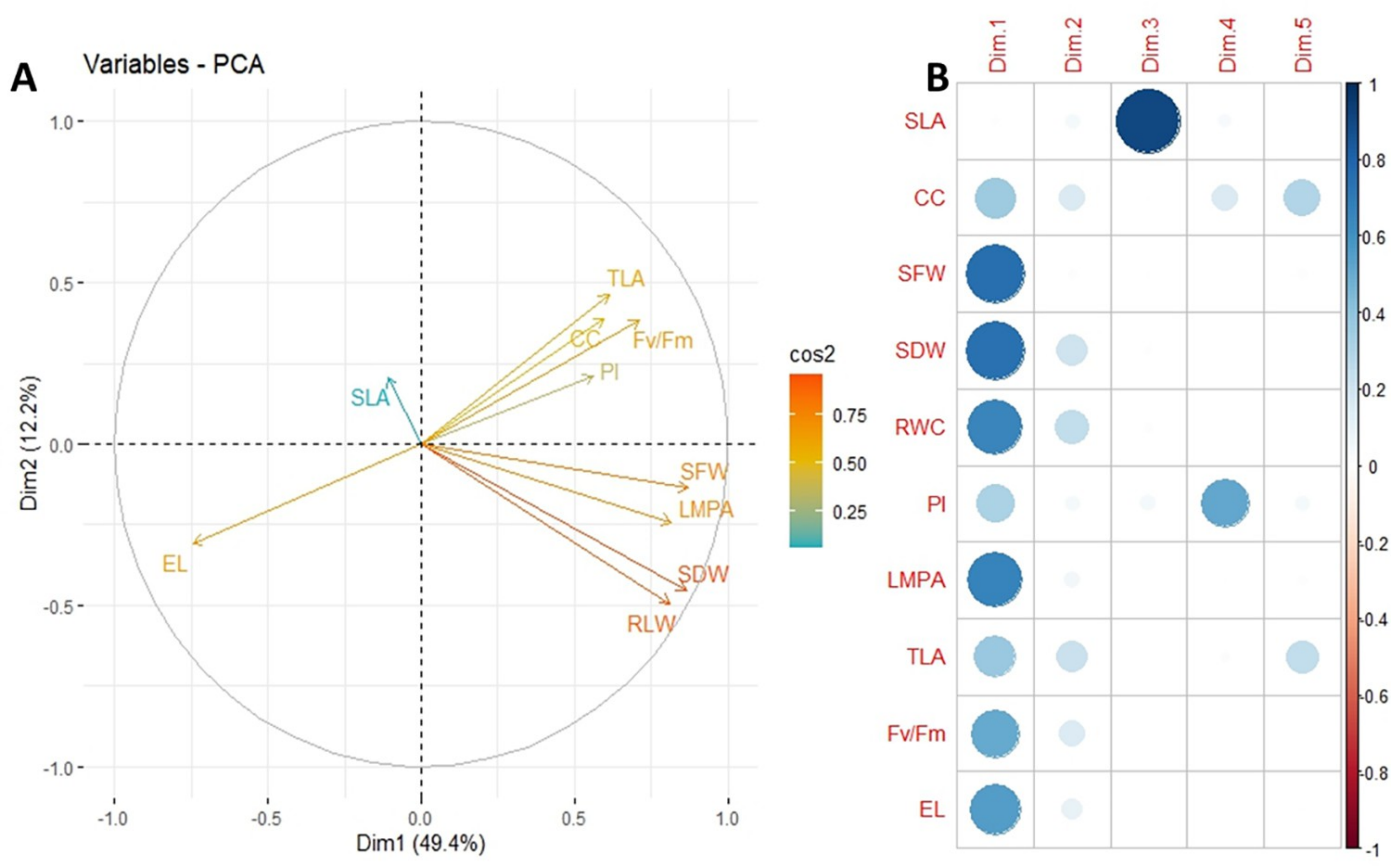
PCA of traits measured from 128 eggplant genotypes screened for heat and drought tolerance at the seedling stage. (A) Loading trait scores on each component of the first five were significant components. (B) Plot of the contribution of variables to the first two principal components for all measured traits. The scale adjacent to the plot indicates the cos2 values of the corresponding variables.

The three principal components (PCs) with an eigenvalue >1 explained 72% of the total variation (Fig 5A). The relative magnitude of PCs 1, 2 and 3 were approximately 49.4, 12.2, and 10%, respectively (Fig 5A). The angle of the trait vector was < 90° for biomass, relative water content, Fv/Fm ratio, and chlorophyll content, suggesting a positive association and independency among these traits. However, the vector angle was > 90° for electrolyte leakage and most measured traits, except for specific leaf area (Fig 5A). In decreasing order, shoot dry weight, relative water content, shoot fresh weight, Fv/Fm ratio and chlorophyll content contributed to the variation observed in PC1 and 2, respectively. In contrast, electrolyte leakage negatively resolved on PC1 (Fig 5A and 5B).

#### 3.5.3 Cluster analysis

**Fig 6:**
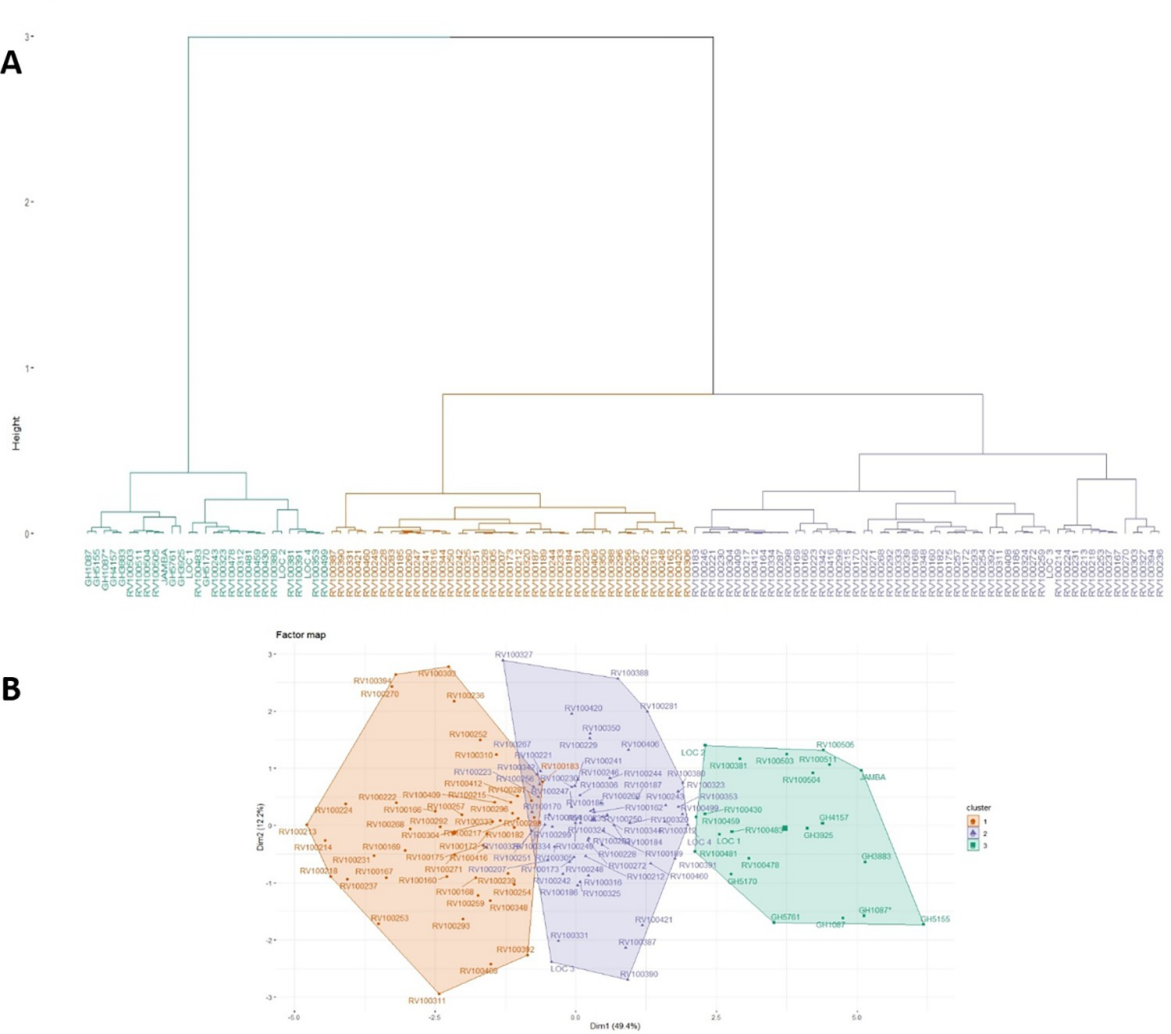
(A) Clustering patterns of traits among the 128 eggplant genotypes. Orange, violet, and green represent Clusters I, II, and III, respectively. Clustering was performed using Ward’s hierarchical approach based on the minimum variance linking method with Euclidean distance as the similarity measure. (B) Factor map showing the contribution of genotype to the observed variation.

The dendrogram from the cluster analysis of 128 African eggplant genotypes revealed three (3) distinct clusters, of which 43, 56, and 29 belonged to groups I, II, and III, respectively (Fig 6A). Genotypes were not categorised based on the country of origin; however, genotypes from Ghana were distributed in cluster III, except for RVI00242 and RV100323, originating from Tanzania and RV100312 from Senegal. The majority of cluster I members, including RV100256, RV100212, RV100283, RV100228, RV100233, RV100296, RV100281, RV100244, RV100250, RV100420, and RV100460, were from West African countries such as Mali, Senegal, Togo, and Nigeria, with genotypes such as RV10089, RV100187, RV100173, and RV100184 from Eastern Africa (Fig 6A). Cluster II genotypes RV100408 and RV100409 originated from India and France, respectively, whereas the RV100166, RV100167, RV100168, RV100169, and RV100416 were from Tanzania, with the majority of members from West Africa (Fig 6A). Generally, cluster membership varied significantly in contributing to the measured traits (Fig 6B). The genotypes in cluster III were characterised by high mean relative water content, biomass, chlorophyll content, Fv/Fm ratio, and lower electrolyte leakage. Members of cluster II were characterised by intermediate Fv/Fm ratio values, biomass, relative water content, and chlorophyll content (Fig 6B). Conversely, cluster I was characterised by higher electrolyte leakage and specific leaf area, with lower biomass, relative water content, and Fv/Fm ratio (Fig 6B).

## 4.0 Discussion

### 4.1 Genotypic variability under single and combined drought and heat stress

Plants frequently encounter combined heat and drought stress episodes in field conditions [50, 51], synergistically impacting the life cycle [11]. However, most studies in both model and crop plants have focused on the effects of drought or heat on yield as single-stress factors [52], except for a few studies in maize [11], groundnut [53], cotton [17], wheat [54] and *Solanum melongena* [55]. Few studies have investigated the tolerance of African eggplants to abiotic stress [55–57]. Developing a reliable and effective screening tool for cultivar tolerance to abiotic stress is vital for building resilience in eggplant production [58]. Morphophysiological parameters such as leaf area, specific leaf area, Fv/Fm ratio, chlorophyll content, relative water content, electrolyte leakage, and biomass [21, 59] used in previous studies were used to screen for tolerance to single and combined heat and drought stress among African eggplant genotypes. Unlike previous studies, the current study used an improvised locally built growth chamber.

Notably, genotypes with high values in chlorophyll content, Fv/Fm ratio, and relative water content also demonstrated higher biomass production in the present study, as indicated in S2 Table. The observed variation in the studied genotypes [59, 60] may be attributed to their genetic makeup and geological location differences. The wide range of diversity within our genotypes suggests that plant morphophysiology may play an essential role in adaptation. Previous studies have also presented genotypic variation in the morphophysiology of Solanaceae, such as tomato, potato, pepper, and eggplant, under heat and drought stress conditions [61–65]. The mean values recorded in the present study for physiological traits, such as relative water content, chlorophyll content, and Fv/Fm ratio, were similar to those recorded in *Solanum melongena* [55], tomato [66], and Jalapeno pepper [67]. We found that genotypes with high-level electrolyte leakage had low chlorophyll content and inflorescence. These results indicate that sensitive eggplant genotypes may suffer from severe damage to their chloroplast and membrane, leading to reduced photosynthetic and low biomass production [68]. Additionally, we observed a positive correlation between shoot biomass, chlorophyll content, relative water content, Fv/Fm ratio, and performance index under contrasting stress conditions (Fig 5A). However, electrolyte leakage and specific leaf area weight pointed in different directions (Fig 5A), suggesting that these morphophysiological traits may be reliable indicators of tolerance to abiotic stress as reported in previous studies in lentils, cotton, chicken pea, tomatoes, and other crops [17, 55, 69, 70]. The significant variations among the screened genotypes can be used to inform selection for improved stress tolerance.

Eggplants are considered tolerant to drought stress (Diaz-Perez & Eaton, 2015). Still, the current study shows that their tolerance to abiotic stress varies depending on the variety, growth stage, intensity, and frequency of stress. The stress response also varies among African eggplant genotypes under heat and drought conditions. Some genotypes, like RV100503, LOC 2, RV100481, GH5761, and RV100430, maintained higher relative water content under single and combined heat and drought stress. Still, the relative water content reduction rate was more pronounced for genotypes under single drought stress, except for RV100187, RV100249, RV100257, and RV100241 (S3 Table). Previous research has found that when plants are subjected to drought and heat stress, the most significant factor that emerges is the hindrance in water use efficiency, which varies among varieties and cultivars [71, 72]. Tolerable genotypes decrease stomatal density, thereby minimising water loss and maintaining internal water balance to cope with these conditions [73]. As a result, genotypes with higher relative water content may possess adaptation or protective mechanisms that help them prevent and avoid the adverse effects of single and combined stressors, thus maximising their photosynthetic efficiency [74].

An increase in electrolyte leakage under stress indicates membrane injury, which reflects the membrane’s stability [75]. Many plant stressors, such as drought and heat, initially attack cell membranes and turgor [76]. The effect is more pronounced under combined heat and drought than the individual effects of these stressors in plants like *Artemisia sieberi alba* [77], potato [78], eggplant [12], and tomatoes [79]. In this study, the genotypes RV100213, RV100228, RV100239, RV100241, RV100242, RV100388, JAMBA, RV100478, RV100483, RV100421, GH1087, GH1087*, GH4157, and LOC 3 had low electrolyte leakage under combined heat and drought compared to drought stress (S3 Table). The genotypes maintained their membrane integrity, an adaptation crucial for plants’ abiotic stress tolerance [80]. The contrasting response of genotypes to single and combined stress underscores the importance of diverse morpho-phenotypic traits in screening populations for identifying useful germplasms for breeding [81]. The results suggest that certain plants may prioritise one acclimation/adaptation strategy over another, blend both, or employ a new strategy [82]. However, the exact choice of acclimation/adaptation strategy during stress combinations may be affected by the intensity of each stress and could vary among genotypes. It is well acknowledged that the effect of drought on membrane integrity is more pronounced than that of heat in eggplants [12] and winter wheat [83]. Thus, genotypes such as GH5761, JAMBA, GH1087, GH5155, RV100511, RV100505, RV100478, RV100503, GH5170, LOC 3, RV100390, RV100380, RV100408, and RV100207, which displayed low electrolyte leakage under drought stress when compared to heat stress, might be exploited in breeding.

Chlorophyll fluorescence analysis using the Fv/Fm ratio and performance index is an excellent tool for evaluating plant photosynthetic performance. It has been proven to be a reliable and fast method for assessing plant tolerance to abiotic stresses [84, 85]. Lower Fv/Fm ratio values (less than 0.83) are observed in plants exposed to biotic or abiotic stress, which reduces their ability to quench energy through photoinhibition [21]. In the current study, tolerant genotypes with Fv/Fm ratio between 0.80 and 0.83 (JAMBA, LOC3, RV100164, RV100169, LOC3, RV100483, GH5155, RV100430, GH1087, GH1087*, RV100388, RV100387, RV100391, etc.) maintained their photosynthetic apparatus better under control treatment. These also experienced less damage under single and combined heat and drought stress. They may have evolved protective mechanisms to prevent damage from high temperature or drought stress and maximise their photosynthetic capacities. Variation in the Fv/Fm ratio and performance index under drought and heat conditions has been reported in crops such as maize [86], wheat [87], tomatoes [88], and okra [89].

Single and combined heat and drought stressors adversely affect biomass production in plants such as tomatoes [90], cabbage [91], and soybeans [92]. Here, genotypes such as RV100503, RV100409, and RV100256 had the highest shoot dry weight production under heat stress conditions compared to the control, drought, and a combination of heat and drought (S3 Table). Our findings indicate that relatively heat-tolerant genotypes showed less membrane damage and improved biomass production than the heat-sensitive genotypes similar to lentils [93] and chickpeas [94]. Combined heat and drought stress accelerates senescence, and causes decreased biomass in numerous crops such as lentil [95], maize [96], and potato [78], as observed in sensitive genotypes such as RV100160, RV100164, RV100167, and RV100183, which recorded low biomass under combined heat/drought compared to control and single stress factors in the present study (S3 Table). In contrast, higher biomass was observed under combined heat and drought for genotypes LOC 3, RV100504, Jamba, GH, RV100185, RV100210, and RV100211 relative to the single effect of drought.

### 4.2 Drought impacts eggplant morphophysiology considerably compared to heat

In response to single heat and drought stress, drought stress had a more significant effect on the morphophysiological traits of eggplant than heat stress, suggesting that the morphophysiology of African eggplant is more sensitive to drought stress than episodes of heat stress. Drought stress resulted in a 24% increase in electrolyte leakage compared to control (Fig 2A). Extensive membrane damage in electrolyte leakage is attributed to the substantial reduction in relative water content, which was 31% more under drought conditions than in the control. The negative association observed between relative water content and electrolyte leakage under heat and drought conditions (S2 Fig) further supports the notion that drought triggers membrane damage, leading to increased ion release due to damaged cell integrity. This observation is consistent with earlier studies that have discussed the harmful impact of drought relative to heat stress on relative water content and electrolyte leakage in potatoes [78], hot peppers, and tomatoes [93], as well as the more prominent drought-induced decrease in plant water potential status in *Phaseolus vulgaris* [97]. A low relative leaf water content of approximately 50% corresponds to severe drought stress under field conditions [98]. In the present study, drought stress might have developed quickly compared to field conditions. Some of Ghana’s major vegetable growing areas have very sandy soils [99], where drought may similarly develop quickly in days.

The intense magnitude of drought stress on the Fv/Fm ratio, chlorophyll content, and performance index under drought conditions was observed in the present study, indicating that drought adversely affects plant physiology by altering photosynthetic efficiency and other functional traits (Fig 2C, 2D and 2G) compared to heat stress. Compared to heat stress, drought caused 15%, 1%, and 25% increases in Fv/Fm ratio, chlorophyll content, and performance index, respectively. Drought alters eggplant’s photosynthetic efficiency and metabolism, probably due to disturbances in chloroplast structural integrity and increased chlorophyll denaturation [100]. Decreased stomatal conductivity during drought and heat stress is considered an adaptive mechanism to reduce water loss through transpiration; however, the trade-off between transpiration and internal temperature, in the long run, could significantly disrupt photosynthetic metabolism and damage cellular structures [101–103]. The biomass production significantly decreased under all stresses, but biomass was more inhibited under drought than heat stress (Fig 2E and 2F). Our data showed that under drought, most photosynthetic traits, including the Fv/Fm ratio, performance index, and chlorophyll content, were all decreased, which reduced the overall net photosynthesis and biomass production of drought-stressed plants. The intercorrelation between biomass and photosynthetic parameters under drought stress conditions (Fig 5A) explains the marked reduction in photosynthetic efficiency observed under such conditions, compared to heat stress alone. Our findings confirm previous research that found a significant decrease in shoot biomass under drought in tomato cultivars compared to heat and control treatments [104].

Although drought and heat are typically linked, heat stress intensifies the negative impacts of drought on plant physiology. Consequently, while it is essential to disaggregate the effects of drought and high temperature on plant productivity, the interactions between these two stressors will complicate this effort. The observed multi-trait effect from drought and heat stress points to a broad spectrum and, possibly, the catastrophic impact of these stresses on plant performance [105]. These stress factors denature protein constituents of the cell membrane and make structures more prone to rupture, often increasing membrane fluidity and ion leakage. Ultimately, this leads to inhibiting various cellular mechanisms [106].

### 4.3 Effects of coexisting heat and drought stress on morphophysiological traits

When multiple stressors affect plants simultaneously, the effect can be additive (combined stress = sum of single stress), synergistic (combined stress > sum of single stress), or antagonistic (combined stress < sum of single stress) [107]. In the present study, the combined stressors significantly impacted the morphophysiological parameters of eggplant, and the result was antagonistic for many traits (Fig 2A–2I). For example, compared to the control, there was a 59% higher electrolyte leakage if the solitary effects of heat and drought stress were summed up. However, the combined effect of heat and drought stress was only 34% higher than that in control. Similarly, the heat and drought stress combination antagonistically affected photosynthetic traits such as Fv/Fm ratio, performance index and chlorophyll content (Fig 2C, 2D and 2G). For example, when heat and drought stress were applied individually, the sum of their effect on chlorophyll content was approximately twice the effect when they were imposed together (Fig 2G). The antagonistic effect of multiple stressors on the morphophysiology `of eggplant reported in the present study is consistent with previous studies, which have also shown that various stressors can have antagonistic effects on traits such as gas exchange, radial growth, and biomass parameters in Pinus sylvestris [108], maize [96], potatoes [78], tomatoes [106], lentils [95], and wheat [11].

We used a simple strategy with only two stressor combinations, which may not reflect the various stressor combinations in the plant’s environment. Zandalinas and Mittler [107] noted that the effect of applied stresses on plants could be synergistic or antagonistic based on the intensity and plant species. Our study showed that the response depends on abiotic stress type, intensity, and duration (Figs 3 and 4). We observed antagonistic effects of heat and drought, which likely reflect their role in stomatal regulation. Drought stress causes stomatal closure to prevent water loss, while increased leaf temperature and heat stress increase transpiration due to a higher vapour pressure deficit between the leaf and air. It’s more likely that when these events occur during drought and heat stress combination, drought pathways overcome heat pathways [107], preventing the full effect of heat stress from being fully expressed in the African eggplant. While these interactions reflect the difficulty in any attempt to distinguish between the impact of heat and drought stress on the morpho-physiology of plants, it also confirms the rationale behind quantifying heat stress with plant or leaf temperature. We screened plants at the juvenile stage, but observing how these two stresses interact in plants that grow to maturity under combined drought and heat conditions would be attractive. Sinha et al. (2022) [109] recently reported a new plant adaptation strategy termed ‘differential transpiration’. Although stomata close during drought and heat stress, plants that employ ‘differential transpiration’ open the stomata on their flowers to maintain transpiration and cool their reproductive tissues.

### 4.4 Low-cost and rapid screening for stress tolerance

The significance of high throughput plant screening and phenotyping has been emphasised [110, 111]. Despite the progress in screening for drought and heat stress in plants, several challenges hinder such studies. The entry-level costs of screening platforms, including large infrastructures and modern heat and growth chambers, have been prohibitive. Equipment cost, for example, is an integral part of the phenotyping process and has often been the most prohibitive part of phenomics in many resource-poor laboratories. The price of climatic test chambers, often used for temperature and humidity testing, temperature cycling, and thermal shock testing, varies depending on size, brand, and complexity. However, excellent and efficient models could cost tens of thousands of dollars. Many research institutions, particularly resource-poor ones, do not have the investment budget to implement robust and high throughput screening approaches even when the technical personnel are available. The present study built an improvised growth chamber that cost just about US$2000 and could screen hundreds of plants during the study period. We designed an ambient and heat growth chamber using locally available materials (Fig 1A) to screen resilient eggplant genotypes to heat, drought, and concurrent heat and drought stress. This low-cost system is noteworthy because reducing the cost of research on genotype-phenotype relationships with an aim toward crop improvement will help researchers in disadvantaged areas improve crop yield without substantial investments in technology and expertise and contribute to global food security [112]. Moreover, the critical criteria for developing rapid screening methods are that the technique must be able to evaluate plant performance at critical stages of development, be inexpensive, and screen many plants in as short a time as possible [113].

Our improvised setup delivered the targets of a low-cost phenotyping system. However, the values obtained for the coefficient of variation (CV), a statistic for comparing variates free from scale effects, were significant, particularly for shoot and leaf-related traits (Table 1). The CV’s magnitude typically results from the complex interplay of genetics, environmental conditions, and stochastic events. A CV exceeding 30% may indicate problems in the data, possibly emanating from sampling variation or flawed data recording [114]. Even so, large CVs in certain traits within a plant population may suggest substantial individual variability for those specific traits, which can be exploited in breeding programmes. While we cannot entirely discount some inherent but inexplicable problems in the data, we believe that the CVs of >20 recorded for leaf and shoot-related traits point to variability among individuals for those specific traits or are just reflective of the small values recorded for these traits at the seeding stage. Accordingly, the results show the setup can measure variation among genotypes, growth chamber, and their interaction with measured traits. This suggests that the structure and method adopted for the study provided a cost-effective, rapid, and reliable means for large-scale screening of germplasm for climate resilience in resource-poor jurisdictions. As we advance, the improvised platform’s precision will be tested with data from conventional growth chambers and different plant species.

## 5.0 Conclusion

Heat and drought are two significant abiotic stress factors that often co-occur and have a significantly detrimental effect on the growth and productivity of crop plants compared to each factor individually. In this study, we hypothesised that heat and drought-tolerance eggplant genotypes can be identified at the seedling stage using a high-throughput technique with locally constructed ambient and heat growth chambers. This method can potentially be a cost-effective, reliable, and accurate way to phenotype abiotic tolerance among many eggplant accessions. The genotypes exhibited a wide range of variation for most traits measured in this study and can be utilised in breeding for heat and drought tolerance. Specifically, the genotypes RV100503, GH5155, GH1087*, GH3883, JAMBA, RV100504, RV100505, RV100478, RV100390, RV100511, RV100392, LOC 3, and LOC 1 displayed tolerance under single and combined heat and drought stress and could provide a relevant gene pool for breeding. These results suggest that chlorophyll fluorescence parameters (Fv/Fm ratio and performance index), relative leaf water content, electrolyte leakage, chlorophyll content, and biomass parameters are valuable traits for establishing diversity among genotypes under single and combined heat and drought stress. However, it should be noted that these results only provide preliminary indications of stress tolerance and may not capture the full range of plant responses under field conditions. Therefore, validation and field trials are crucial for confirming the performance of the identified genotypes under realistic stress scenarios.

## Supporting information

S1 Fig Mean temperature and relative humidity for locally constructed ambient and heat chamber during the screening period. (DOCX)

S2 Fig Correlation matrix among measured morphophysiological traits among seedlings of African eggplant genotypes cultivated under drought, heat and combined heat and drought conditions. (DOCX)

S1 Table Characteristics, source and origin of eggplant genetic materials. (XLSX)

S2 Table Genotypic variation in the morphophysiology of 5-weeks old African eggplant cultivated under contrasting stress conditions. (XLSX)

S3 Table Morphophysiological response of African eggplant to heat, drought and combined heat and drought stress. (XLSX)
