# Supplementary material for: Rapid and low-cost screening for single and combined effects of drought and heat stress on the morpho-physiological traits of African eggplant (Solanum aethiopicum) germplasm
Source: PLoS One. 2024 Jan 30;19(1):e0295512. doi: 10.1371/journal.pone.0295512 (PMC10826938; doi:10.1371/journal.pone.0295512)
Supplement: S1 Fig — (DOCX) [file pone.0295512.s001.docx]

Figure S1: Mean temperature and relative humidity for locally constructed ambient and heat chamber during the screening period.
